# Extra-Virgin Olive Oil and Its Minor Compounds Influence Apoptosis in Experimental Mammary Tumors and Human Breast Cancer Cell Lines

**DOI:** 10.3390/cancers14040905

**Published:** 2022-02-11

**Authors:** Maite Garcia-Guasch, Mireia Medrano, Irmgard Costa, Elena Vela, Marta Grau, Eduard Escrich, Raquel Moral

**Affiliations:** 1 Department of Cell Biology, Physiology and Immunology, Faculty of Medicine, Universitat Autònoma de Barcelona, Bellaterra, 08193 Barcelona, Spain; mariateresa.garcia.guasch@uab.cat (M.G.-G.); Mireia.Medrano@uab.cat (M.M.); Elena.Vela@uab.cat (E.V.); gr.mecm@uab.cat (M.G.); Eduard.Escrich@uab.cat (E.E.); 2 Department of Pathology, Corporació Parc Taulí-UDIAT, 08208 Sabadell, Spain; icosta@tauli.cat

**Keywords:** breast cancer, experimental mammary tumors, high-fat diets, apoptosis, cell death, olive oil, hydroxytyrosol, oleuropein, luteolin

## Abstract

**Simple Summary:**

Breast cancer is a disease influenced by dietetic factors, such as the type and amount of lipids in a diet. In this work, we aimed to elucidate the different effects of two high-fat diets on the histopathological and molecular characteristics of mammary tumors in an experimental model. Animals fed with a diet high in extra-virgin olive oil (EVOO), compared to those fed with a diet high in seed oil, developed tumors with less aggressiveness and proliferation. Tumor molecular analyses of several cell death pathways also suggested an effect of EVOO in this process. In vitro experiments indicated the role of EVOO minor compounds on the effects of this oil. Obtaining insights into the influence and the mechanisms of action of dietary compounds are necessary to understand the relevance that dietetic habits from childhood may have on health and the risk of disease.

**Abstract:**

Breast cancer is the most common malignancy among women worldwide. Modifiable factors such as nutrition have a role in its etiology. In experimental tumors, we have observed the differential influence of high-fat diets in metabolic pathways, suggesting a different balance in proliferation/apoptosis. In this work, we analyzed the effects of a diet high in n-6 polyunsaturated fatty acids (PUFA) and a diet high in extra-virgin olive oil (EVOO) on the histopathological features and different cell death pathways in the dimethylbenz(a)anthracene-induced breast cancer model. The diet high in n-6 PUFA had a stimulating effect on the morphological aggressiveness of tumors and their proliferation, while no significant differences were found in groups fed the EVOO-enriched diet in comparison to a low-fat control group. The high-EVOO diet induced modifications in proteins involved in several cell death pathways. In vitro analysis in different human breast cancer cell lines showed an effect of EVOO minor compounds (especially hydroxytyrosol), but not of fatty acids, decreasing viability while increasing apoptosis. The results suggest an effect of dietary lipids on tumor molecular contexts that result in the modulation of different pathways, highlighting the importance of apoptosis in the interplay of survival processes and how dietary habits may have an impact on breast cancer risk.

## 1. Introduction

Breast cancer is the most common cancer in women worldwide, with high incidence, prevalence, and mortality rates [1]. This neoplasia has a multifactorial etiology, in which diet, as an environmental factor, has an important role. Epidemiological and especially animal studies have reported an influence of diet and dietary fat on breast cancer [2,3]. In general, saturated fatty acids and n-6 polyunsaturated fatty acids (PUFA) have shown a promoting effect on breast cancer, whereas n-3 PUFA, as well as the ratio of n-3/n-6 PUFA intake, showed an inverse association with breast cancer risk [3]. Regarding monounsaturated fatty acids (MUFA), evidence is still uncertain, and experimental studies have described effects ranging from a weak promoting effect to a protective effect on mammary carcinogenesis [4]. In this sense, the Mediterranean diet, of which a main source of fat is olive oil (rich in the n-9 MUFA oleic acid), is considered a healthy dietary pattern, with beneficial effects on the risks of cardiovascular diseases, obesity, and cancer. Epidemiological studies have demonstrated that adherence to this diet is associated to a reduction of overall and cancer mortality [5,6,7].

Obtaining insight into the effects that diet and nutrition have on cancer is inherently complex, as nutritional factors would exert a subtle and probably long-term influence in such a multifactorial disease. However, it is of great importance to determine these effects and the mechanisms involved, since exposure to these nutritional factors can occur from childhood and throughout life. Animal experimental models are essential in diet and cancer research, considering that nutrients and other dietary components may play a physiopathological role, interacting at many molecular and cellular levels. We have previously demonstrated in the 7,12-dimethylbenz[α]anthracene (DMBA)-induced model of mammary cancer in rats a differential effect of high-fat diets, with a diet high in n-6 PUFA having a clear tumorigenesis-stimulating effect, while there was a weak influence of the isocaloric diet high in extra-virgin olive oil (EVOO; rich in MUFA and phenolic bioactive compounds) [4,8,9]. These effects were associated with several mechanisms, such as changes in growth and sexual maturation, hormonal status, mammary gland biology, and tumor molecular modifications, including transcriptomic changes modulating the function of cell death-related genes [4,8,9,10,11,12,13]. We have also recently reported that this high-EVOO diet, in comparison to the high n-6 PUFA diet, induced modifications in tumor metabolic characteristics suggesting a higher glucose uptake, glycolysis rate, and oxidative phosphorylation [13]. Although such metabolic features have been associated with tumor progression [14,15,16], these tumors displayed a less aggressive phenotype, highlighting that those metabolic characteristics without the context of other pathways, such as proliferation or apoptosis, may not reflect tumor malignancy.

Apoptosis is a programmed and ordered cell death process occurring in many physiological and pathological conditions. The mechanisms of programmed cell death are complex and driven by many pathways. Caspases are the main involved proteins, as they act as initiators and executioners, and can be activated by three pathways: the extrinsic, the intrinsic, and the endoplasmic reticulum pathway. Apoptosis can also be induced in a caspase-independent manner [17,18]. Reduced cell death, or its resistance, has a key role in carcinogenesis. Cancer cells can disrupt the balance of pro-apoptotic and anti-apoptotic proteins, reduce caspase function, and impair death receptor signaling in order to avoid programmed cell death and sustain uncontrolled growth [18]. Targeting apoptosis mechanisms is important in cancer treatment and the basis of many therapeutic approaches [19]. From the field of nutrition, several works are addressing the effect of some phytochemicals on apoptosis as potential prevention and treatment agents in cancer [20], although several factors, such as their bioavailability, are currently important limitations. On the other hand, few studies have addressed the effect of diet on tumor apoptosis in vivo. Understanding the mechanisms by which diet can influence cancer risk is needed to establish prevention and treatment strategies. Thus, the aim of this work is to elucidate the effect that the high-fat diets, high in a seed oil (rich in n-6 PUFA) or in extra-virgin olive oil (rich in oleic acid and bioactive compounds), and with different timings of dietary interventions, may have on the interplay among metabolism, proliferation, and apoptosis, with a special focus on the different apoptotic pathways (Figure S1). In vitro analysis in different human breast cancer cells with different fatty acids (oleic acid, linoleic acid) and minor compounds (hydroxytyrosol, oleuropein, and luteolin) were also carried out to acquire insight into the molecular basis of the influence of EVOO on breast cancer.

## 2. Materials and Methods

### 2.1. Animals and Experimental Design

All animals received humane care under an institutionally approved experimental animal protocol, following the legislation applicable in this country. Female Sprague–Dawley rats (stain Crl: OFA (SD), *n* = 100) were obtained from Charles River Lab (L’Arbresle Cedex, France) at 23 days of age. Three semisynthetic diets were designed: the low-fat diet (LF; 3% corn oil -*w*/*w*-), the high corn oil diet (HCO; 20% corn oil) and the high extra-virgin olive oil diet (HEVOO; 3% corn oil + 17% EVOO). The definition, preparation, and suitability of the experimental diets were previously described in studies [8,21,22]. Animals were distributed in five groups depending on the diet and the timing of dietary intervention (*n* = 20 each group): the LF diet from weaning (LF group), the HCO diet from weaning (HCO group) or after induction (LF-HCO group), and the HEVOO diet from weaning (HEVOO group) or after induction (LF-HEVOO group). At 53 days of age, mammary tumors were induced by oral gavage with one single dose of 5 mg of DMBA (Sigma-Aldrich, St. Louis, MO, USA) dissolved in corn oil (25 mg DMBA/kg body weight). From day 74 onwards, animals were monitored weekly for the appearance of mammary tumors. At 236–256 days of age, animals were euthanized by decapitation, selecting the rats that were in the diestrus phase of the estrous cycle, and between 10:00 h and 13:00 h to avoid circadian rhythm variability (Figure S2). Tumors were removed, a portion of each was fixed in 4% formalin for histopathological analysis, and the rest was flash-frozen and stored at −80 °C for molecular analysis.

Tumor histopathology characterization was determined by applying the Scarff–Bloom–Richardson grading method (SBR3; scoring 1–3), and also the method adapted to rat mammary carcinomas that we have previously described (SBR11; scoring 3–11) [23], with the highest categories indicating the most aggressive tumors. SBR scores include differentiation pattern, nuclear pleomorphism, and mitotic index. Number of mitoses were determined in 10 high power fields (HPF) and classified into five categories [23]. Only confirmed mammary adenocarcinomas were included in this study.

### 2.2. Protein Extraction

Tumor samples were homogenized in an extraction buffer (50 mM Tris–HCl pH 7.2, 250 mM sucrose, 2 mM EDTA, 1 mM EGTA, 5 mM MgCl_2_, 50 μM NaF, 100 μM Na_3_VO_4_, 10 μL/mL protease inhibitor cocktail (Sigma-Aldrich, St. Louis, MO, USA), 10 mM β-mercaptoethanol, and 1% Triton X-100) for total protein extraction. For fractioned extracts, samples were cold centrifugated at 105,000× *g* for one hour to separate the soluble fraction (corresponding to cytosol) from the particulate fraction, which was further resuspended in extraction buffer with 0.1% Triton X-100 and centrifuged at 105,000× *g* (supernatant corresponding to the membranous fraction containing all membranes). Mitochondrial extract was obtained from homogenized samples by centrifuging at 1500× *g* to separate nuclei, followed by supernatant centrifuging at 10,000× *g* to obtain precipitated mitochondria. Protein quantification was determined by Lowry’s method using the commercial DC Protein Assay Kit (Bio-Rad, Hercules, CA, USA) (cytosolic, mitochondrial, and membrane).

### 2.3. Western Blot

The different protein extracts (10–20 µg) were subjected to SDS-PAGE electrophoresis on an acrylamide gel (7.5% or 12% Mini-Protean TGX Stain-Free Gels, Bio-Rad, Hercules, CA, USA) and transferred to a PVDF membrane with Trans-Blot Turbo Transfer System (Bio-Rad). Membranes were blocked with 5% BSA or 5% skimmed milk in TBS-0.1% Tween for 1 h at room temperature and incubated with primary antibody overnight at +4 °C. The primary antibodies and dilutions used were: AIF (1:2000), APAF-1 (1:2000), Bak (1:2000), Bax (1:2000), Caspase-3 (1:2000), Caspase-8 (1:1000), Caspase-9 (1:1000), Cytochrome C (1:500), p53 (1:500), TNFR1 (1:500), TRADD (1:1000), and XIAP (1:500) from Cell Signaling (Leiden, Holland); Bcl2 (1:1000), Bid (1:3000), EndoG (1:1000), FADD (1:1000), and Fas (1:500) from Santa Cruz Bioechnologies (Dallas, TX, USA); TRAFf2 (1:3000) from ThermoFisher Scientific (Waltham, MA, USA); and Caspase-12 (1:2000) from Abcam (Cambridge, UK). Membranes were incubated with the secondary antibody (anti-mouse or anti-rabbit IgG peroxidase-conjugated) for 1 h at room temperature and developed with Luminata Forte Western HRP Substrate (Merk Millipore, Burlington, MA, USA) luminogen for 3–5 min. Proteins bands were visualized using the Chemidoc XRS+ hardware associated with Image Lab Software 5.1-Beta (Bio-Rad). Densitometric values were normalized with the total protein loaded [24] and relativized to an internal control sample loaded in duplicates in all blots.

### 2.4. Cell Culture Treatment

For in vitro analyses, we used the MCF-7 cell line, representing the molecular subtype of human breast cancer Luminal A, and MDA-MB-231 cells, representing the triple-negative subtype. Cell lines were obtained from the American Type Culture Collection (ATCC; LGC Standards, Middlesex, UK). MCF-7 cells were grown in EMEM (Gibco, Thermo Fisher Scientific, Waltham, MA, USA) and supplemented with 10% fetal bovine serum (FBS; Gibco) and 0.01 mg/mL of insulin. MDA-MB-231 cells were grown in DMEM (Gibco) and supplemented with 10% FBS. Cells were maintained at 37 °C in a humidified atmosphere of 95% air and 5% CO_2_. The cell cultures were subjected to Mycoplasma screen tests periodically.

For fatty acid treatments, oleic acid- or linoleic acid-albumin from bovine serum (Sigma-Aldrich, Merck KGaA, Darmstadt, Germany) were used at 0 µM (control), 1 µM, 10 µM, 100 µM, or 1 mM concentration. All the solutions contained 0.1 mg/mL of BSA (Sigma-Aldrich).

For treatments with EVOO minor compounds, we used hydroxytyrosol (PHL80152, Sigma Aldrich) at 0 µM (control), 100 µM, 250 µM, and 400 µM; oleuropein (12,247, Sigma Aldrich) at 0 µM (control), 10 µM, 30 µM, and 50 µM; and lutein (L9283, Sigma-Aldrich) at 0 µM (control), 5 µM, 10 µM, and 30 µM [25,26,27]. All the solutions contained 0.1% of DMSO.

### 2.5. Viability Assays

For analysis of cellular metabolic activity as an indicator of cell viability and proliferation, we used MTT colorimetric assay based on the reduction of thiazolyl blue tetrazolium bromide (Sigma-Aldrich) to formazan. Briefly, MCF-7 cells were seeded in 96-multiwell plates at 6 × 10^3^ cells/well in 100 μL of complete EMEM (Gibco) medium with 5% FBS. MDA-MB-231 cells were seeded in 96-multiwell plates at 5 × 10^3^ cells/well in 100 μL of complete DMEM (Gibco) medium with 5% FBS. After 24 h, the medium was replaced with fresh one containing the specific treatment and incubated for 24 to 72 h. MTT reagent was added to each well (10 μL/well of MTT solution at 5 mg/mL in PBS) and incubated 2 h at +37 °C. Then, formazan crystals were solubilized in 100 μL of 100% DMSO and absorbances were read at 570 nm. Relative cell viability was calculated as the ratio of absorbance of treated samples and control samples.

### 2.6. Determination of Apoptotic Cells by Flow Cytometry

MCF-7 and MDA-MB-231 cells were seeded 24 h before treatment in 12-multiwell plates at 2 × 10^5^ cells/well in 1 mL of complete EMEM and DMEM (Gibco) medium, respectively, with 5% FBS. Cells were treated with the specific concentration of oleic acid, linoleic acid, hydroxytyrosol, oleuropein, or lutein for 24, 48, and 72 h. Cells were trypsinized, washed in PBS, and labelled with Annexin-V-Fluorescein and Propidium iodide (PI) using an Annexin-VFLUOS staining kit (Inmunostep, Salamanca, Spain) according to the manufacturer instructions. Then, data were acquired on a FACSCalibur flow cytometer at SCAC Facility, UAB.

### 2.7. Mitochondrial Membrane Potential Assay by JC-1 Staining

MCF-7 cells were seeded 48 h before treatment in 6-multiwell plates at 4 × 10^5^ cells/well and treated for 24, 48, and 72 h with 0 or 400 µM of hydroxytyrosol (Sigma Aldrich). Briefly, cells were collected by trypsinization, stained with JC-1 dye (15 µg/mL), and incubated at room temperature for 15 min at 37 °C in a humidified atmosphere of 95% air and 5% CO_2_. Data were acquired on a FACSCalibur flow cytometer at SCAC Facility, UAB.

### 2.8. Cell Cycle Analysis

MCF-7 cells were seeded 48 h before treatment in 6-multiwell plates at 3 × 10^5^ cells/well, 2.5 × 10^5^ cells/well and 2 × 10^5^ cells/well and treated for 24, 48, and 72 h respectively with 0 or 400 µM of hydroxytyrosol (Sigma-Aldrich). Cells were collected by trypsinization, fixed with 70% ethanol, and stored at −20 °C. On the analysis day, cells were washed with PBS, labelled with 0.5 mL/1 × 10^6^ cells of FxCycle PI/RNse staining solution (ThermoFisher Scientific), and incubated at room temperature for 30 min. Data were acquired on a FACSCalibur flow cytometer at SCAC Facility, UAB.

### 2.9. Statistical Analysis

Statistical analyses were performed using R Deducer. The statistical test to be used was determined depending on the distribution of each variable (Shapiro–Wilk test) and the equality of variances among groups (Levene’s test). Data from tumors usually do not follow a normal distribution, and the non-parametrical Kruskal–Wallis test—Wilcoxon method was used. Quantitative parametrical data were analyzed with one-way ANOVA test (*t*-test equal variance) and paired *t*-test. For qualitative data (distributions and percentages) the Pearson’s Chi-squared test was used. The level of significance was established at *p* < 0.05. For in vivo results, distribution of data is represented using box plots. Box indicates interquartiles (IQR; first quartile Q1, second quartile = median, and third quartile Q3); whiskers extend from maximum (Q3 + 1.5 × IQR) to minimum (Q1 − 1.5 × IQR); dots represent outliers; and cross indicates the mean value.

## 3. Results

### 3.1. The High-Corn Oil Diet Influenced the Manifestation of the Disease

Carcinogenesis parameters are shown in Table 1. The tumor latency period (days from induction to first palpable malignant tumor) was shorter in the HCO and LF-HCO groups, although differences did not reach statistical significance. On the other hand, the percentage of tumor-bearing animals and total number of tumors were higher in the groups fed the HCO diet in comparison to the LF and HEVOO groups. Tumor multiplicity (mean number of tumors per animal) was also increased in the HCO group in comparison to the LF group.

### 3.2. The High-Corn Oil Diet, but Not the High-EVOO Diet, Had a Clear Effect on Histopathological Malignancy and Proliferation of Tumors

**Figure 1:**
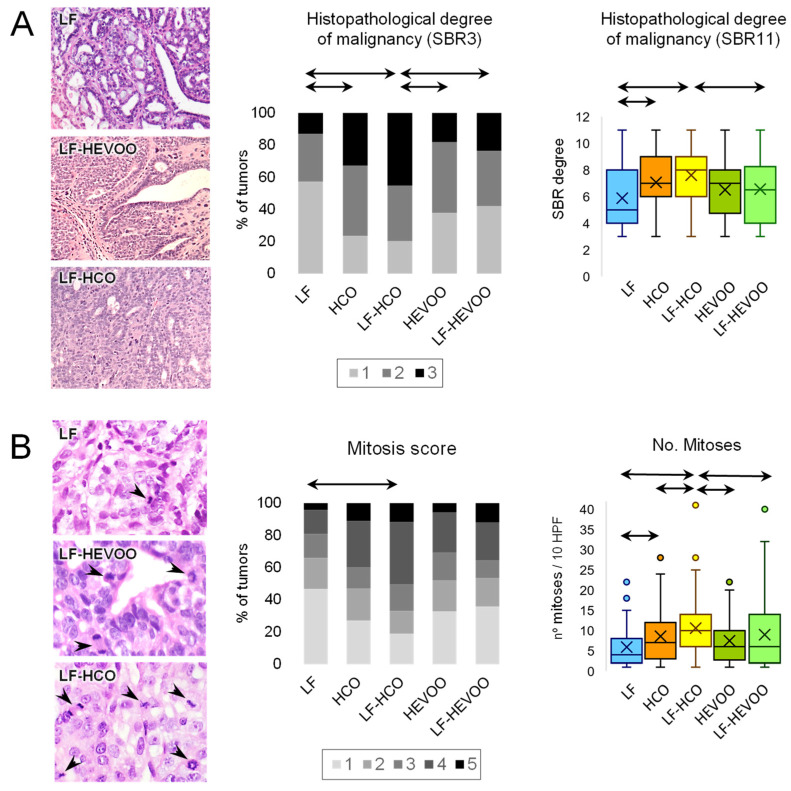
Effect of high-fat diets on the degree of aggressiveness and proliferation of experimental mammary tumors. (**A**) Histopathological classification of tumors. Images show representative DMBA-induced mammary adenocarcinomas with different degree of morphological aggressiveness. Stacked bar chart depicts the distribution of mammary adenocarcinomas of each group according to the Scarff–Bloom–Richardson index, scoring tumors in three categories (SBR3), with the highest representing the most aggressive. Box and whisker plot depicts data of the modified Scarff–Bloom–Richardson index (SBR11) of each tumor. (**B**) Mitosis study. Images show representative determination of mitoses in tumors with different mitosis score. Stacked bar chart depicts the distribution of mammary adenocarcinomas of each group according to the mitosis score. Box and whisker plot depicts data of the number of mitoses in 10 high power field (HPF, 400× magnification) for each tumor. Arrows connecting groups indicate differences statistically significant (*p* < 0.05), Chi-squared test (distribution data: SBR3 and mitosis score), non-parametric Mann–Whitney U test (quantitative data: SBR11 and number of mitoses). LF, *n* = 46; HCO, *n* = 100; LF-HCO, *n*= 87; HEVOO, *n* = 57; LF-HEVOO, *n* = 82. Scale bar.

Results for the morphological degree of tumor aggressiveness and proliferation are shown in Figure 1. Histopathological analyses indicated a higher degree of malignancy (both in SBR3 and SBR11 score) in the groups fed the HCO diet in comparison to the groups fed the control and high-EVOO diets. A histological quantitation of proliferation indicated a higher mitosis score in the high-corn oil diet groups versus control, especially in LF-HCO, and a great number of mitoses in both HCO-fed groups in relation to the control. LF-HCO also showed a great number of mitoses in comparison to the high-EVOO diet groups.

### 3.3. The EVOO-Enriched Diet Promoted the Expression of Extrinsic Apoptosis Pathway Proteins

**Figure 2:**
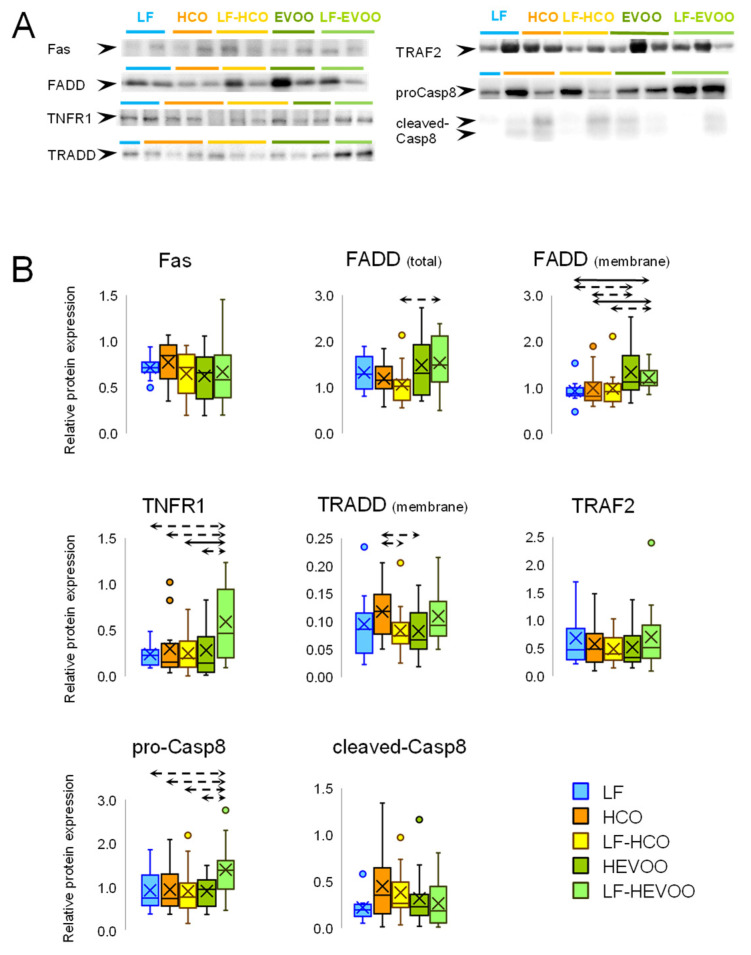
Effect of high-fat diets on the expression of apoptotic extrinsic pathway proteins in experimental tumors. (**A**) Detection of proteins by Western blot in the different groups. (**B**) Box plots represent distribution data for Fas, FADD (total and membrane), TNFR1, TRADD (membrane), TRAF2, and Caspase-8 (pro- and cleaved-Casp8). FADD (membrane) and TRADD (membrane) were detected in the membrane fraction, the other proteins were detected in total protein extract. Box: 25th percentile, median, 75th percentile; whiskers: maximum to minimum; dots: outliers; cross: mean value. Solid arrows connecting groups indicate statistically significant differences (*p* < 0.05), dashed lines indicate differences close to significance (*p* < 0.1), Kruskal–Wallis test.

The protein expression levels of extrinsic pathway-related proteins (Fas, FADD total and membrane fraction, TNFR1, TRADD membrane fraction, TRAF2, pro-Caspase-8, and cleaved-Caspase-8) were studied by Western blot and the results are shown in Figure 2 (Original Western blots in Supplementary material). Changes in protein expression levels were observed in FADD, TNFR1, and pro-Caspase-8 with higher levels in the LF-HEVOO group when compared to LF group. When comparing between high-fat diets, FADD (total and membrane fraction), TNFR1, and pro-Caspase-8 showed higher levels in the LF-HEVOO group versus LF-HCO group, while the HEVOO group showed higher FADD and TRADD in comparison to the HCO group. Higher expression levels of TNFR1 and pro-Caspase-8 were also found in LF-HEVOO versus HEVOO.

### 3.4. The EVOO-Enriched Diet Increased the Expression of Intrinsic Apoptosis Pathway Proteins

**Figure 3:**
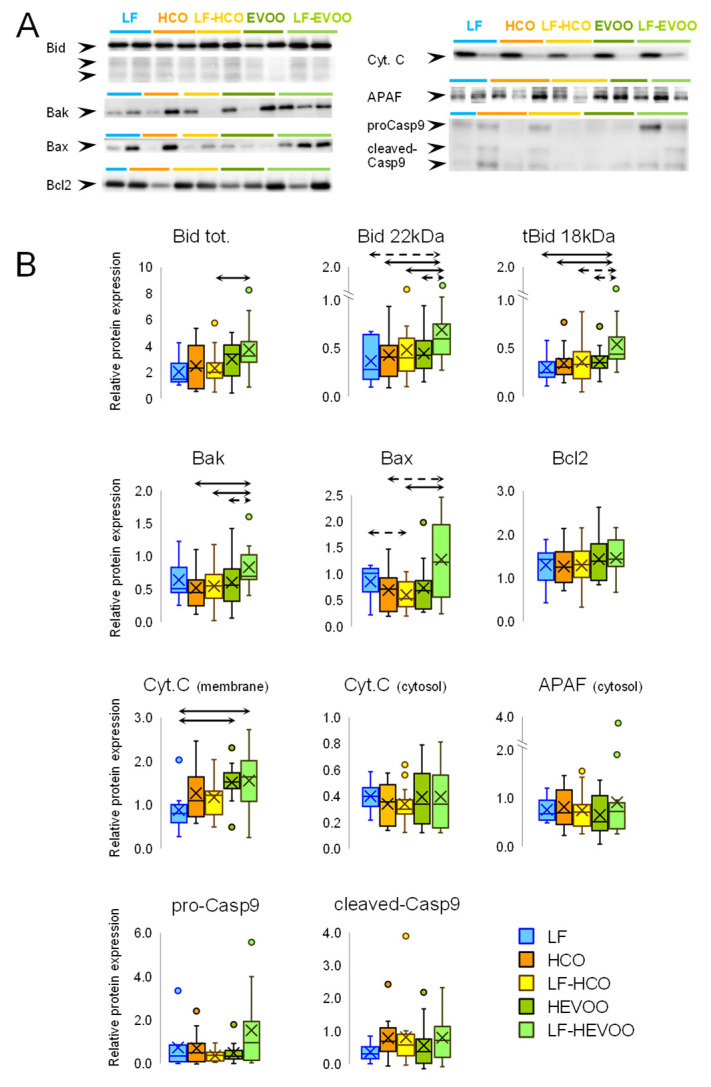
Effect of high-fat diets on the expression of apoptotic intrinsic pathway proteins in experimental tumors. (**A**) Detection of proteins by Western blot in the different groups. (**B**) Box plots represent distribution data for Bid (total and 22 kDa, and the active truncated 18 kDa tBid), Bak, Bax, Bcl2, Cytochrome C (cyt.C) in membrane and cytosol, APAF in cytosol, and Caspase-9 (pro- and cleaved-Casp9). Cyt.c (membrane) was detected in membrane fraction, Cyt.C (cytosol) and APAF (cytosol) were detected in cytosol fraction, the other proteins were detected in total protein extract. Box: 25th percentile, median, 75th percentile; whiskers: maximum to minimum; dots: outliers; cross: mean value. Solid arrows connecting groups indicate statistically significant differences (*p* < 0.05), dashed lines indicate differences close to significance (*p* < 0.1), Kruskal–Wallis test.

We have also studied proteins involved in the apoptotic intrinsic pathway: the pro-apoptotic Bid and the active tBid, Bak, Bax, Cytochrome C in mitochondrial and cytoplasm fraction, APAF, pro-Caspase-9, cleaved-Caspase-9, and the anti-apoptotic Bcl2 (Figure 3 and Original Western blots in Supplementary material). Relative protein expression levels of the total Bid, tBid, and Cytochrome C (membrane fraction) were higher in the LF-HEVOO group than in the LF group. This LF-HEVOO group also showed higher levels of total Bid, tBid, Bak, and Bax when compared to the HCO-fed groups, and those of Bid and Bak when compared to the HEVOO group.

### 3.5. Effect of High-Fat Diets on Endoplasmic Reticulum (ER) Stress-Induced Cell Death and in Apoptosis Pathways Convergence

**Figure 4:**
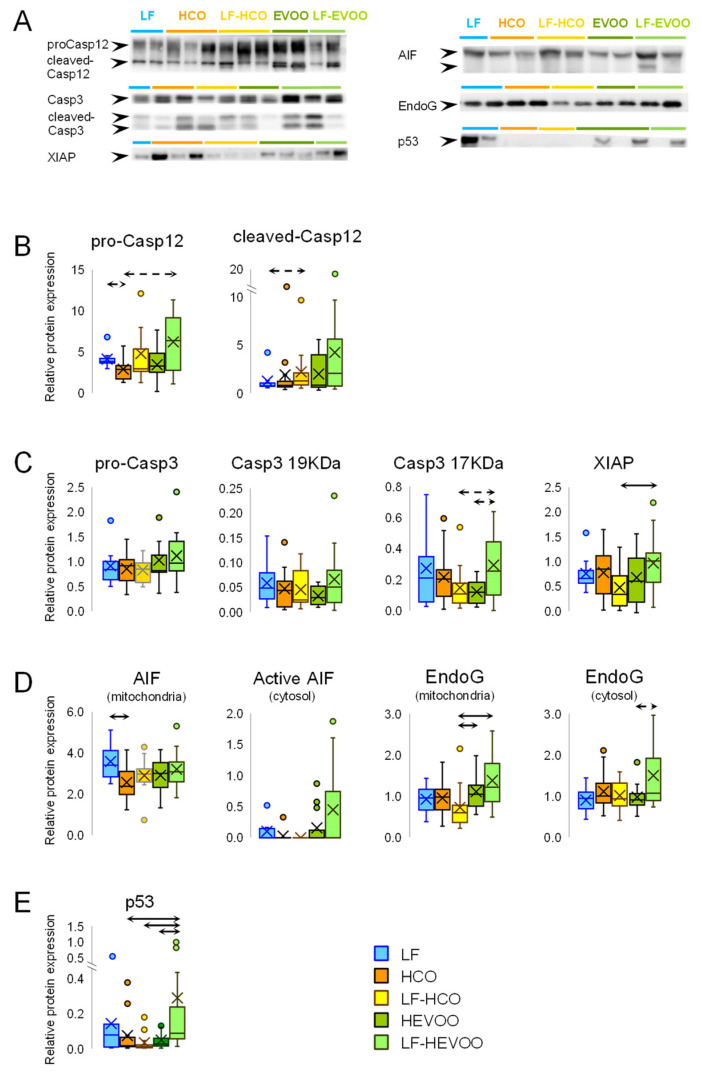
Effect of high-fat diets on the expression of apoptotic proteins in experimental tumors. (**A**) Detection of proteins by Western blot in the different groups. (**B**) ER stress-induced apoptosis protein Caspase-12 (pro- and cleaved-Casp12). (**C**) The key protein in confluence of apoptotic pathways Caspase-3 (pro-Casp3 and the 19 KDa and 17 Da cleaved forms), and its inhibitor XIAP. (**D**) Caspase-independent cell death proteins AIF (total in mitochondria and active cytosolic AIF levels) and EndoG (mitochondria and cytosolic). (**E**) Total protein levels of p53. AIF and EndoG were detected in mitochondria fraction and total protein extract, the other proteins were detected in total protein extract. Box: 25th percentile, median, 75th percentile; whiskers: maximum to minimum; dots: outliers; cross: mean value. Solid arrows connecting groups indicate statistically significant differences (*p* < 0.05), dashed lines indicate differences close to significance (*p* < 0.1), Kruskal–Wallis test.

Results for the Caspase-12 protein, which is involved in ER stress-induced cell death, are shown in Figure 4A and in Original Western blots in Supplementary material. There was a trend in the HCO group to have lower levels of pro-Caspase-12, while a trend to have higher levels of the active cleaved form was observed in the LF-HCO group.

On the other hand, the protein expression levels of pro-Caspase-3 and active Caspase-3 are shown in Figure 4B and in Supplementary material. There was a trend to have increased levels of 17 kDa cleaved-Caspase-3 in the LF-HEVOO group compared to the LF-HCO and HEVOO groups. The Caspase-3 inhibitor, XIAP, was increased in the LF-HEVOO group in comparison to LF-HCO group.

### 3.6. Effect of High-Fat Diets on Caspase Independent Cell Death and on p53

We studied AIF and EndoG as proteins related to caspase-independent cell death (Figure 4C and Original Western blots in Supplementary material). The total AIF (67 KDa) in mitochondria was decreased in the HCO group in comparison to the LF group. Active AIF (57 KDa) in cytosol was detected in a few samples, with a higher frequency (Pearson’s Chi-squared test, *p* < 0.05) in the LF-HEVOO group. EndoG levels in mitochondria were significantly higher in the LF-HEVOO and HEVOO groups compared to the LF-HCO group, while there was a trend toward higher levels of cytosolic EndoG in the LF-HEVOO group.

In relation to p53, total levels were significantly higher in the LF-HEVOO group in comparison to all other high-fat diet groups (Figure 4D and Supplementary material).

### 3.7. Effect of Oleic and Linoleic Acids on Viability and Apoptosis in Breast Cancer Cells

**Figure 5:**
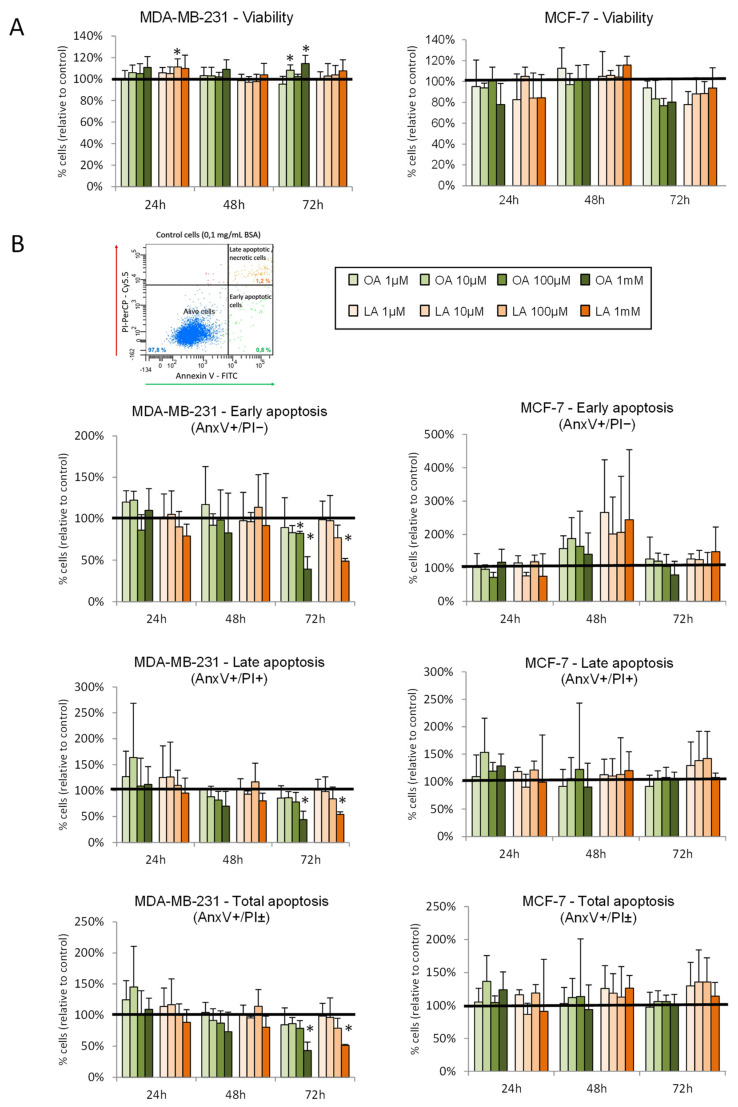
Effects of fatty acid treatments on cell viability and apoptosis in human breast cancer cell lines. (**A**) Cell viability analysis using the MTT assay of MDA-MB-231 and MCF-7 cell lines treated with different doses of oleic acid (OA) and linoleic acid (LA) for 24 h, 48 h, or 72 h. (**B**) Apoptosis analyses by flow-cytometry assay of Annexin V/propidium iodide (PI) double staining. Picture shows a representative image of dot plot diagrams identifying cell population stained with Annexin-V (AnxV, green) and PI (red). For MDA-MB-231 and MCF-7, graphics show the results for cells in early apoptosis (AnxV+/PI−), late apoptosis (AnxV+/PI+), and total apoptosis (AnxV+/PI±), relative to control cells. Mean + standard deviation of three–five independent experiments. *: *p* < 0.05 compared to control cells (0.1 mg/mL of BSA).

MDA-MB-231 and MCF-7 cells were treated with oleic and linoleic acid at different concentrations (1, 10, 100, and 1000 μM) for 24 h, 48 h, and 72 h. As shown in Figure 5A, oleic and linoleic acids had no effects on MCF-7 cell viability. In MDA-MB-231 cells, both fatty acids had a slight stimulating effect compared to the control (increased viability with oleic acid at 10 µM and 1 mM for 72 h, and with linoleic acid at 100 µM after 24 h of treatment). Regarding apoptosis (Figure 5B), no effects on MCF-7 were observed. In MDA-MB-231, both oleic and linoleic acids decreased the percentage of apoptotic cells after 72 h of treatment with the highest doses (Figure 5B).

### 3.8. EVOO Main Polyphenols Affected Cell Viability in Breast Cancer Cells

**Figure 6:**
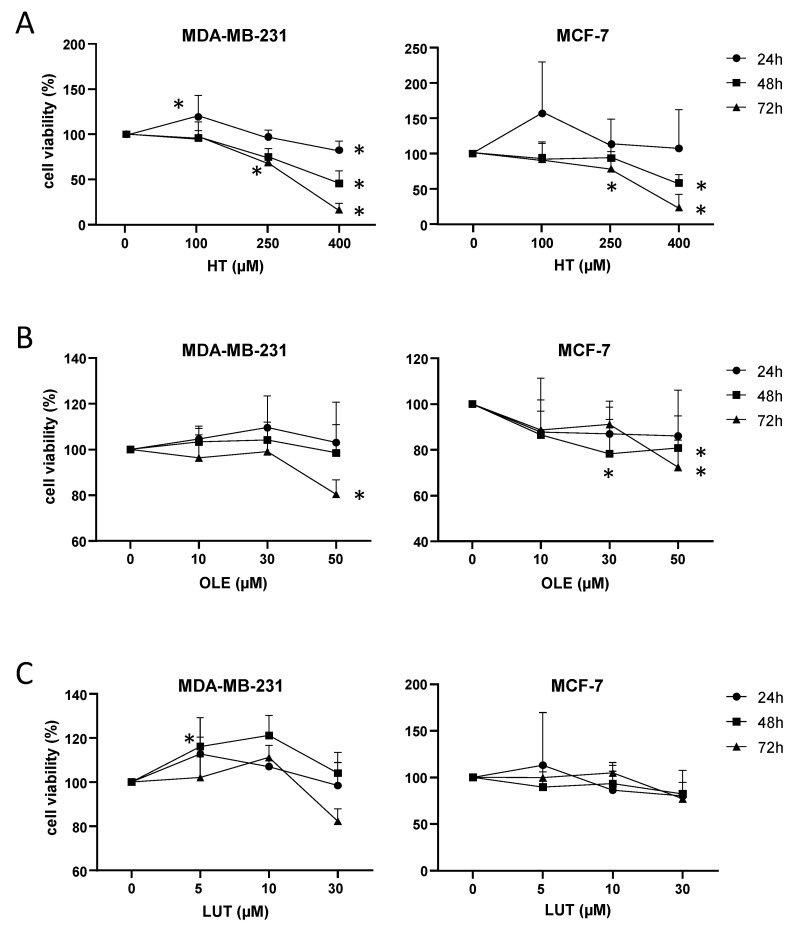
Effect of EVOO minor compounds on cell viability in MDA-MB-231 and MCF-7 cell lines treated with different doses for 24 h, 48 h, or 72 h, relative to control cells (0 µM). (**A**) Hydroxytyrosol (HT). (**B**) Oleuropein (OLE). (**C**) Luteolin (LUT). Mean + standard deviation of three independent experiments. *: *p* < 0.05 compared to control cells (0.1% DMSO-treated).

MDA-MB-231 and MCF-7 cells were treated with different doses of hydroxytyrosol (HT), oleuropein (OLE), and luteolin (LUT) for 24, 48, and 72 h. Figure 6 depicts the comparison of treated cells versus control cells (treated only with 0.1% DMSO). HT had a time- and dose-dependent effect, decreasing cell viability both in MDA-MB-231 and MCF-7 cells. At the highest dose (400 µM), HT decreased viability at 48 h and 72 h in both cell lines. At 72 h, viability was also decreased with 100 μM (MDA-MB-231) and 250 µM (MDA-MB-231 and MCF-7). By 72 h, OLE at 50 µM decreased viability in both cell lines, and in MCF-7 there was also a significant effect at 30 µM after 48 h and 72 h. LUT did not decrease viability at the doses used in these cell lines; we only observed an increase in MDA-MB-231 with 5 µM by 24 h.

A comparison among different doses and times of treatment are indicated in Figure S3. In both lines, cells treated with 400 µM of HT for 48 h and 72 h showed a significant decrease in the cell viability in comparison to cells treated with 100 µM and 250 µM. At 400 µM there were also a time-dependent effect, especially in MDA-MB-231 cells (lower viability at higher times of treatment). MDA-MB-231 cells treated with OLE 50 µM for 72 h had a decreased viability in comparison to cells treated with OLE at 30 µM. The highest concentration of LUT (30 µM) for 72 h also decreased cell viability in comparison to LUT at 5 µM and 10 µM.

### 3.9. Hydroxytyrosol Promoted Apoptosis in Breast Cancer Cell Lines

**Figure 7:**
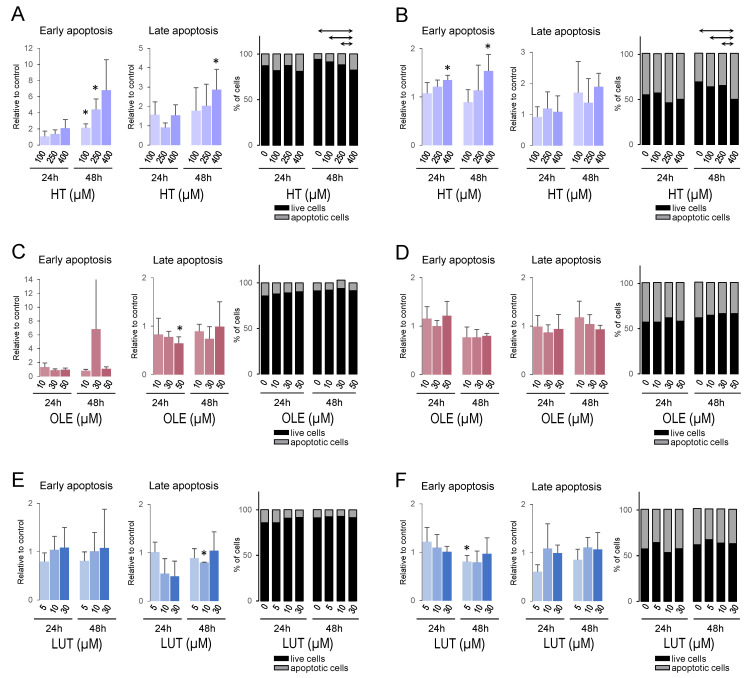
Effect of treatment with HT, OLE, and LUT on apoptosis in MDA-MB-231 and MCF-7 cell lines. Apoptosis analyses by flow-cytometry assay of Annexin V/propidium iodide (PI) double staining in MDA-MB-231 (**A**,**C**,**E**) and MCF-7 (**B**,**D**,**F**) cells treated with different doses of hydroxytyrosol (**A**,**B**), oleuropein (**C**,**D**), or luteolin (**E**,**F**) for 24 h or 48 h. For each treatment and cell line, results show values of early apoptosis compared to control cells (0.1% DMSO-treated cells), values of late apoptosis compared to control cells, and percentage of live/apoptotic cells. Histograms show mean + standard deviation of three independent experiments. *: *p* < 0.05 compared to control cells (0.1% DMSO-treated). Solid lines connecting bars indicate statistically significant differences between doses.

Annexin V/PI staining and flow cytometry were used to evaluate the effect of EVOO polyphenols in cell apoptosis. MDA-MB-231 and MCF-7 cells were treated with increasing doses of HT, OLE, and LUT for 24 h and 48 h. Figure 7 shows results for early apoptosis (annexin positive/propidium iodide negative-labeled cells) and late apoptosis (annexin positive/propidium iodide positive-labeled cells), and a percentage of live and apoptotic cells for each treatment in MDA-MB-231 (Figure 7A,C,E) and MCF-7 (Figure 7B,D,F). HT increased early apoptosis in both cell lines (especially at 48 h in MDA-MB-231, Figure 7A, and at the highest dose after 24 h and 48 h in MCF-7, Figure 7B). The highest dose (400 µM) also increased late apoptosis after 48 h in MDA-MB-231 cells. A comparison of the percentage of live/apoptotic cells among doses showed a significant dose-effect after 48 h in both cell lines, with a higher percentage of apoptotic cells in 400 µM-treated cells compared to all other doses. Few differences were found with OLE and LUT treatments, with no clear trend.

### 3.10. Hydroxytyrosol Did Not Significantly Affect Cell Cycle or Mitochondrial Membrane Potential in MCF-7

**Figure 8:**
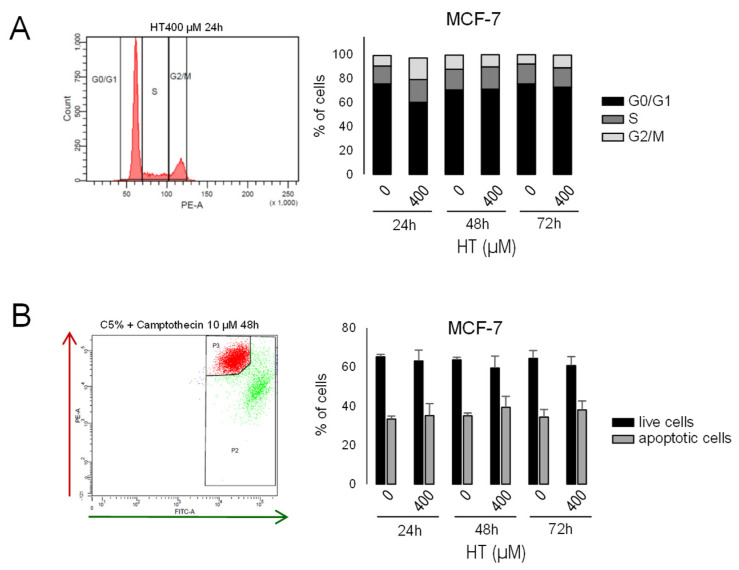
Effect of treatment with HT on cell cycle and membrane depolarization in MCF-7 cells. (**A**) Flow cytometry DNA content analysis of cells stained with propidium iodide (G0/G1 and G2/M phase peaks are separated by the S-phase). Distribution of cells in G0/G1, S, and G2/M in control and 400 µM HT-treated MCF-7 cells of three independent experiments. (**B**) Flow cytometry dot plot showing the gating of JC1 aggregates (live cells, red fluorescence) and JC1 monomer (low mitochondrial membrane potential, apoptotic cells, and green fluorescence) populations. Percentage of live/apoptotic cells in control and 400 µM HT-treated MCF-7 cells of three independent experiments.

Further analysis on the effect of HT was carried out on MCF-7 cells. We studied by flow cytometry the cycle of cells treated with HT at 400 µM for 24, 48, and 72 h (Figure 8A and Figure S4A). On the other hand, mitochondrial membrane potential was analyzed by staining with JC-1, a cationic dye indicator of potential-dependent accumulation in the mitochondria (Figure 8B and Figure S4B). No statistically significant differences were observed in the HT treatment.

## 4. Discussion

In this work, we aimed to analyze the effects of two high-fat diets, rich in n-6 PUFA or rich in EVOO, on the characteristics of the aggressiveness and proliferation of DMBA-induced mammary tumors, and to obtain insight into the molecular mechanisms of such effects. We have previously demonstrated a different influence of these diets on the manifestation of experimental tumors, clearly stimulating in the case of the high n-6 PUFA diet, and with the high-EVOO diet having a weak influence [4,9,10,11,12]. Here, we have observed that tumors from both groups fed with the high n-6 PUFA diet, from weaning (HCO group) or after induction (LF-HCO group), displayed the highest tumor histopathological grade, indicating more aggressiveness [23]. The number of mitoses and the mitosis score were also higher in such groups. On the other hand, tumors from groups fed with the high-EVOO diet were not significantly different to those from the control low-fat group. Considering that high-fat intake is associated with an increase in breast cancer risk [28,29], and that both experimental high-fat diets were isocaloric, our results suggest a specific beneficial effect of the EVOO that can partially counteract the detrimental impact of an excessive intake of fat.

The effects that dietary lipids may exert on mammary tumorigenesis are likely driven by multiple and complex molecular mechanisms acting simultaneously. We have recently demonstrated that these high-fat diets induced metabolic changes depending on the type and timing of dietary intervention [13]. The high-EVOO diet, in comparison to the high n-6 PUFA diet, induced modifications suggesting an increase in the glucose uptake and glycolysis, pentose phosphate pathway (PPP), tricarboxylic acid cycle, and energy dissipation by UCP2. Although these pathways have been reported as deregulated in cancer [14,15,16], the clinical and morphological characterization of tumors showed that such changes were not associated to aggressiveness. Our data also pointed out that the relevance of metabolic changes depend on the interplay with other signaling pathways, such as apoptosis [30]. In this sense, in a previous transcriptomic analysis, we have observed different effects of these high-fat diets on the expression profiles of genes related to metabolism, proliferation, and apoptosis pathways [9]. Thus, we have analyzed in the experimental tumors the effect of diets on the protein levels of several pathways leading to cell death: intrinsic, extrinsic, ER stress-induced, and caspase-independent (Figure S1). The results showed that several pro-apoptotic proteins increased in the tumors from animals fed the EVOO diet, especially in the LF-HEVOO group. In this group, we found higher levels of the proteins of the extrinsic apoptosis pathway TNFR1, FADD, and pro-Caspase-8, although there were no differences in active-Caspase-8 levels. On the other hand, several mitochondrial proteins with a role in the intrinsic apoptosis pathway were also increased (tBid, Bax, Bak, and Cytochrome C), and no differences were found in cytoplasmic Cytochrome C or active-Caspase-9. Caspase-12, involved in ER stress-induced apoptosis, showed a tendency to be increased in the LF-HEVOO group, and the active-Caspase-12 increased in the LF-HCO group. Caspase-3, the key protein in the confluence of the apoptotic pathways, showed no marked changes, which could be related to the increase in its inhibitor XIAP. Despite this, a statistical trend towards higher levels of 17 kDa cleaved-Caspase-3 was found in the LF-HEVOO group. Regarding the caspase-independent pathway, membrane EndoG levels were increased in such EVOO diet groups, also showing a trend towards higher levels of cytosolic EndoG. Although few samples showed active AIF, the percentage of tumors with detectable cytoplasmatic levels of active AIF was significantly higher in the LF-HEVOO group. Finally, we also observed significantly higher levels of the p53 protein in this group. Taken together, all these results raise several hypotheses. One is that, given the complexity and heterogeneity of the experimental tumors, the set of results is much more informative than the specific proteins, so these data suggest greater cell death due to the effect of the high-EVOO diet through several pathways. Another possibility is that despite observing increases in various pro-apoptotic proteins, anti-apoptotic proteins are also upmodulated, thus some effectors are not increased. Additionally, little changes in these proteins are enough to contribute to the aggressive phenotype in the context of other pathways, and methodological issues cannot be ruled out. In any case, the analysis of all these proteins and pathways suggests that cell death could be increased in the LF-HEVOO group (which displays a tendency towards greater levels of cleaved-Caspase-3, active AIF, EndoG, and p53), which is consistent with the histopathological characteristics of this group. The fact that the same results have not been found in the group fed this diet from weaning (HEVOO) is consistent with the greater volume of their tumors [9,12]. It is also concordant with metabolic changes observed in the HEVOO group, i.e., an increase in glycolysis and PPP, which has been associated with a decrease in apoptosis [13]. This metabolic profile favors NADPH generation, which may lead to caspase inhibition [31,32].

Further analyses were performed with the components of the oils in different cell lines characteristic of different molecular subtypes of human breast cancer: MDA-MB-231, representing the triple-negative molecular subtype, and MCF-7, representing the Luminal A subtype. MCF-7 is a non-aggressive, hormone receptor-positive, and non-invasive cell line, while the MDA-MD-231 cell line is an estrogen receptor-, progesterone receptor-, and HER2-negative, highly proliferative and invasive cell line [33]. In accordance, the proliferation rate was higher in MDA-MD-231 cells, while the percentage of cells in apoptosis was lower in comparison to MCF-7 cells. We analyzed cell viability and apoptosis in cells treated with the main fatty acids of the two oils used in vivo: oleic acid (main fatty acid of EVOO) and linoleic acid (main fatty acid of corn oil). In MDA-MB-231, but not in MCF-7, both fatty acids had a slight effect on viability, while the higher doses after 72 h decreased apoptosis. It is likely that the effect of oleic acid depends on time, dose, and the type of cell. It has been reported in other cell lines that oleic acid treatment induced apoptosis [34,35], but it has also been shown to increase proliferation, migration, or invasion in cancer cells [36,37]. It has been suggested that the heterogeneity in fatty acid metabolism may be at the basis of the different response of cell lines to fatty acid treatments [38]. On the other hand, we analyzed the effect of some of the main minor compounds of EVOO: the secoiridoid oleuropein (OLE), its metabolite hydroxytyrosol (HT), and the main flavonoid luteolin (LUT) [39]. HT and OLE had a dose- and time-dependent effect, decreasing cell viability on both MDA-MB-231 and MCF-7 cells. HT also stimulated apoptosis in both cell lines, while little effect was observed with OLE and LUT. The preliminary results with HT have not shown changes by this polyphenol in cell cycle or membrane potential, the latter suggesting that apoptosis would not be led from the intrinsic pathway, although further analyses are needed to elucidate the involvement of the different cell death pathways. The results with hydroxytyrosol and oleuropein agree with those described by other authors in relation to their anti-proliferative and apoptotic properties [40,41]. On the contrary, with the doses used, and in these cell lines, we did not find a significant effect of luteolin on viability and apoptosis, although other authors reported anti-inflammatory and antioxidant effects [42], and tumor suppressor activity in DMBA-induced tumors [43,44]. Recently, a mitogenic effect of oleic acid has also been observed in colon cancer cells, while the effect of several minor compounds of EVOO was the opposite [45], which highlights the importance of the specific composition of the oils consumed and the complexity of the interaction of nutritional factors in vivo.

All this data suggest that dietary fats have a molecular effect on tumors that influences their histopathological degree and proliferation through various mechanisms, specific and nonspecific, that interact with each other, creating a myriad of responses. The results are consistent, for example, with changes in the membrane composition of tumors as a function of the type of lipid consumed [46,47], concomitantly with a specific effect of different minor compounds, which would have effects in different signaling pathways heterogeneously. Thus, we have observed increases in the EVOO groups in the proteins involved in the extrinsic apoptosis pathway, in which changes in microdomains can have a role, and in intrinsic and caspase-independent pathways, which can be also related to the effect of EVOO minor compounds. In this sense, we have previously observed changes in p21Ras location and activity, which has been related to an oleic acid-enrichment of membranes that can modify microdomains and thus several signaling pathways [12,46]. In addition, our in vitro results, both in hormone receptor-positive (representing Luminal A human breast cancer) and hormone receptor-negative (representing the triple-negative subtype) cells, suggest that HT and OLE are able to influence different breast cancer cell lines through estrogen receptor-independent pathways. In the case of the rodent DMBA model, the mammary tumors developed reflect the most common less-aggressive human breast cancer, being hormone-sensitive, low proliferative, and with no overexpression of HER2 [23,48]. In any case, data in the literature suggest several mechanisms of action of EVOO minor compounds, including hormone receptor-dependent and -independent effects [49]. Many EVOO components have shown anticancer activities by modulating gene expression, the direct activation/inhibition of proteins and enzymes, or antioxidant activity, which may in turn regulate apoptosis [49,50].

The results also showed the effect of the timing of dietary intervention. High-fat diets were administered from weaning (groups HCO and HEVOO), or after induction (groups LF-HCO and LF-EVOO). Both groups fed the high-EVOO diet had lower clinical and histopathological levels of malignancy than high corn oil diet groups. However, the LF-HCO group had the highest degree of clinical and morphological malignancy, while LF-EVOO is the group in which more differences in cell death proteins have been found. In previous works, we have observed that the high-fat diets, especially the high corn oil diet, advanced the puberty onset [10]. Thus, with an accelerated puberty maturation, the observed difference in carcinogenesis yield can be related to the different degree of differentiation that the mammary gland was at during the time of the carcinogenic insult. If the mammary gland of groups LF-HCO and LF-HEVOO were less differentiated at the time of induction, the gland could be more sensitive to the influence of the high-fat diets, clearly carcinogenesis-promoting in the case of the high corn oil diet (resulting in the highest degree of malignancy in the LF-HCO group), and with an effect of EVOO on cell death pathways (more evident in the LF-EVOO group). Moreover, we also previously observed an increase in body weight and mass if the high corn oil diet is administered from weaning [10]. Considering that advanced puberty onset and obesity are known risk factors of breast cancer, our results highlight the importance of acquiring healthy choices from an early age.

Despite the variability of some results, the group of tumors from animals fed with the high-EVOO diet have different characteristics, such as low proliferation, modifications in several cell death pathways, and the reported metabolic changes [13]. However, neither of these pathways, without the interrelation of all networks, characterized by itself the clinical manifestation and the degree of tumor aggressiveness in vivo. In vitro analyses support an effect of EVOO on proliferation and apoptosis, with minor compounds such as hydroxytyrosol and oleuropein playing a role. In vivo, it is likely that the fatty acids also have a role as carriers of minor compounds and/or in the absorption of such compounds, in addition to the reported importance of the ratio between fatty acids on breast cancer risk [51,52]. Taking into account the tumor-promoting influence of a high intake of total fat [28,29], our investigations support the beneficial effect of the moderate intake of extra-virgin olive oil (which contains all the bioactive elements) on the risk of breast cancer. The complexity of this disease suggests that the effects of nutritional factors are subtle, heterogeneous, and probably in the long-term, but potentially relevant, especially considering that exposure, depending on dietary habits, can occur chronically throughout life. Thus, more investigation on the effects and mechanisms of these modifiable factors is needed, which can provide important scientific bases for dietary recommendations in health and disease.

## 5. Conclusions

The results reported show the specific effect of the type of dietary lipid in breast carcinogenesis with the diet high in n-6 PUFA diet but not the high-EVOO diet, promoting mammary tumors with a high degree of histopathological malignancy and proliferation. This is the first study reporting that EVOO increased proteins leading to different cell death pathways in experimental mammary tumors. In vitro results support that the potential beneficial effect of EVOO is mainly elicited by its minor components, such as hydroxytyrosol (decreased tumor cell variability and increased cell death), and highlight the importance of specific food compositions (e.g., different effects of olive oil, extra-virgin olive oil, and oleic acid-enriched seed oil) in breast cancer prevention and treatment strategies.

## Figures and Tables

**Table 1:** Effect of high-fat diets on DMBA-induced tumorigenesis in rats.

Parameter	LF	HCO	LF-HCO	HEVOO	LF-HEVOO
Latency period (days, mean ± SE)	98.6 ± 11.9	81.8 ± 9.9	76.4 ± 9.3	86.4 ± 10.3	94.8 ± 6.8
Tumor incidence (%)	80	100 ^a,b^	100 ^a,b^	75	85
(Tumor-bearing rats/total)	16/20	20/20	20/20	15/20	17/20
Total number of tumors	46	100 ^a,b^	87 ^a^	57	83 ^a^
Tumor multiplicity (no. tumors/animal, mean ± SE)	2.3 ± 0.5	5.0 ± 0.7 ^a^	4.4 ± 0.9	2.9 ± 0.8	4.2 ± 1.0

^a^: *p* < 0.05 in comparison to control LF group; ^b^: *p* < 0.05 in comparison to HEVOO group. Continuous variables (latency time, tumor multiplicity): Kruskal–Wallis test. Categorical variables (tumor incidence, total number of tumors): Pearson’s Chi-squared test.

## Data Availability

Molecular data from Western blots generated in this study are included in Supplementary Materials.

## Supplementary Materials

The following supporting information can be downloaded at: https://www.mdpi.com/article/10.3390/cancers14040905/s1. Figure S1: cell death signaling pathways; Figure S2: experimental design; Figure S3: cell viability of MDA-MB-231 and MCF-7 cell lines treated with EVOO minor compounds; Figure S4: original plots of the in vitro analysis of cell cycle and apoptosis by flow cytometry; Original Western blot data: original blots and densitometry analyses for all the proteins studied.
