# Supplementary material for: Extra-Virgin Olive Oil and Its Minor Compounds Influence Apoptosis in Experimental Mammary Tumors and Human Breast Cancer Cell Lines
Source: Cancers (Basel). 2022 Feb 11;14(4):905. doi: 10.3390/cancers14040905 (PMC8870719; doi:10.3390/cancers14040905)
Supplement: Supplementary file 1 [file cancers-14-00905-s001.zip › cancers-1403976-supplementary.pdf]

cancers

MDPI

Supplementary Materials

Extra-Virgin Olive Oil and Its Minor Compounds Influence Apoptosis in Experimental Mammary Tumors and Human Breast Cancer Cell Lines

Maite Garcia-Guasch [1,†], Mireia Medrano [1,†], Irmgard Costa [2], Elena Vela [1], Marta Grau [1], Eduard Escrich [1] and Raquel Moral [1,*]

[1] Department of Cell Biology, Physiology and Immunology, Faculty of Medicine, Universitat Autònoma de Barcelona, 08193 Bellaterra, Barcelona, Spain; MariaTeresa.Garcia.Guasch@uab.cat (M.G.-G.); Mireia.Medrano@uab.cat (M.M.); Elena.Vela@uab.cat (E.V.); gr.mecm@uab.cat (M.G.); Eduard.Escrich@uab.cat (E.E.)
[2] Department of Pathology, Corporació Parc Taulí-UDIAT, 08208 Sabadell, Barcelona, Spain; icosta@tauli.cat
* Correspondence: Raquel.Moral@uab.cat
† These authors contributed equally to this work.

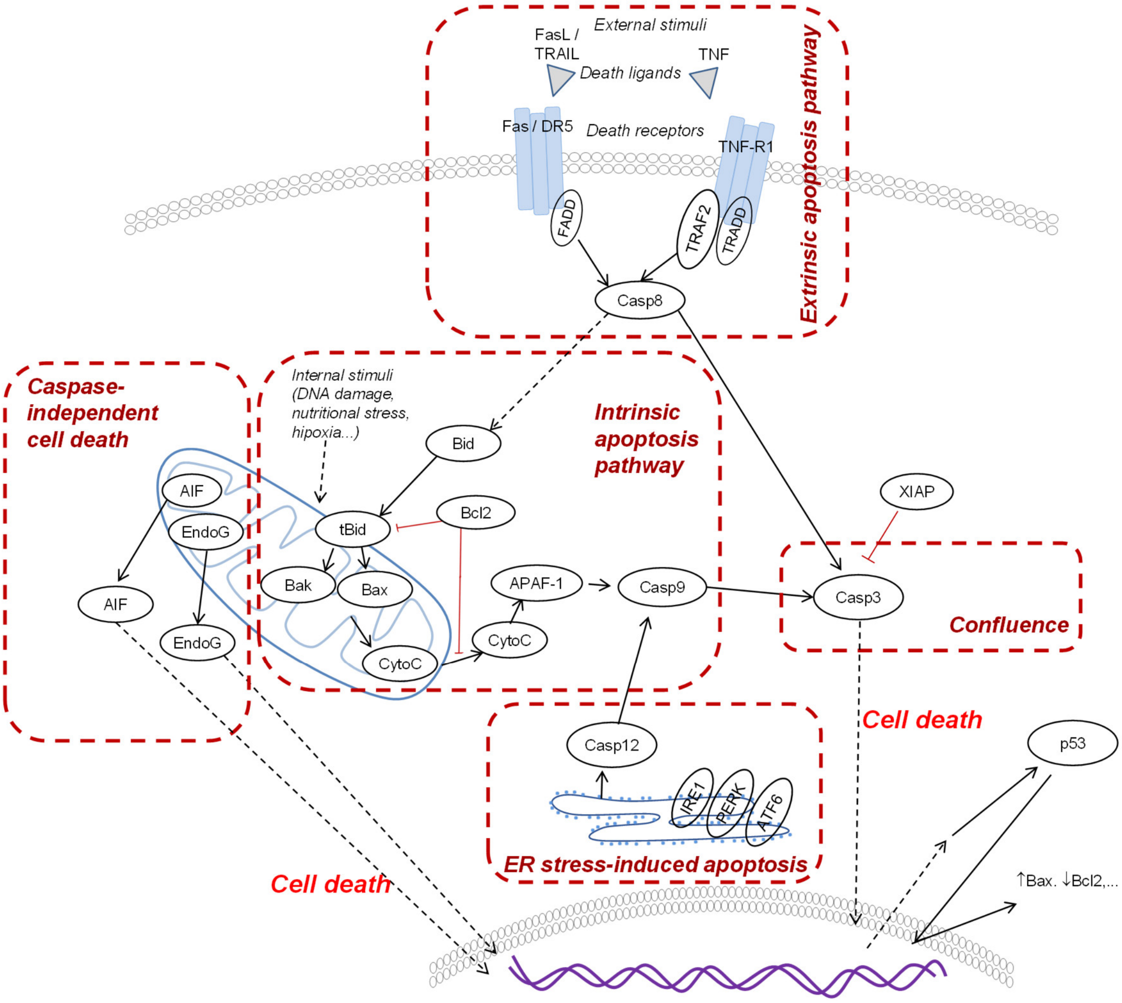

Figure S1. Different cell death signaling pathways: extrinsic apoptosis, intrinsic apoptosis, confluence between extrinsic and intrinsic pathways, ER stress-induced apoptosis, and caspase-independent cell death.

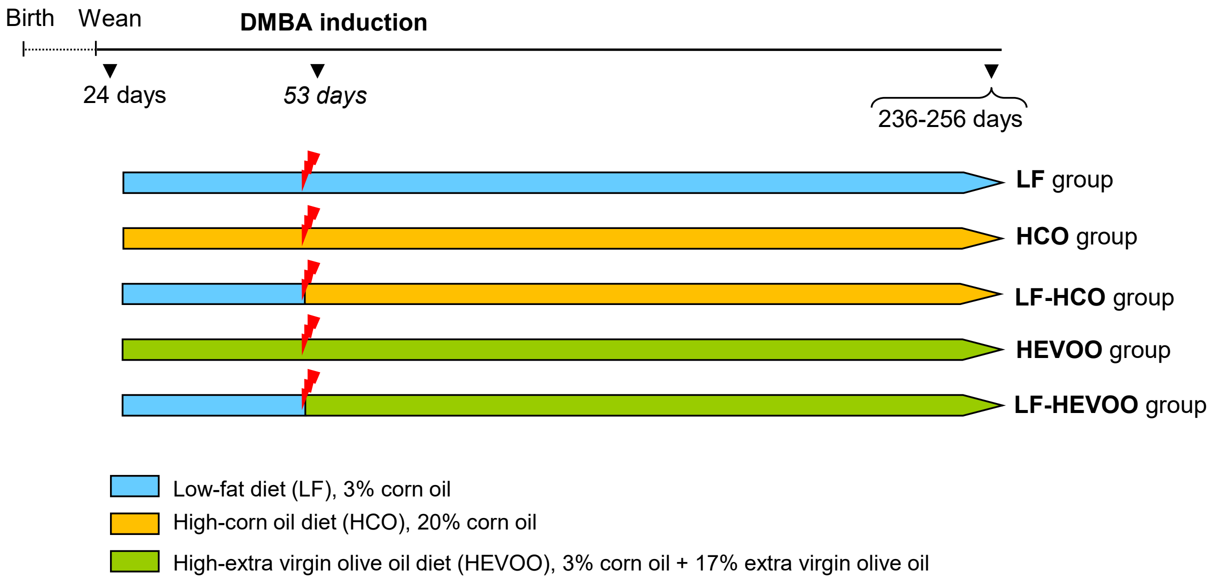

Figure S2. Experimental design.

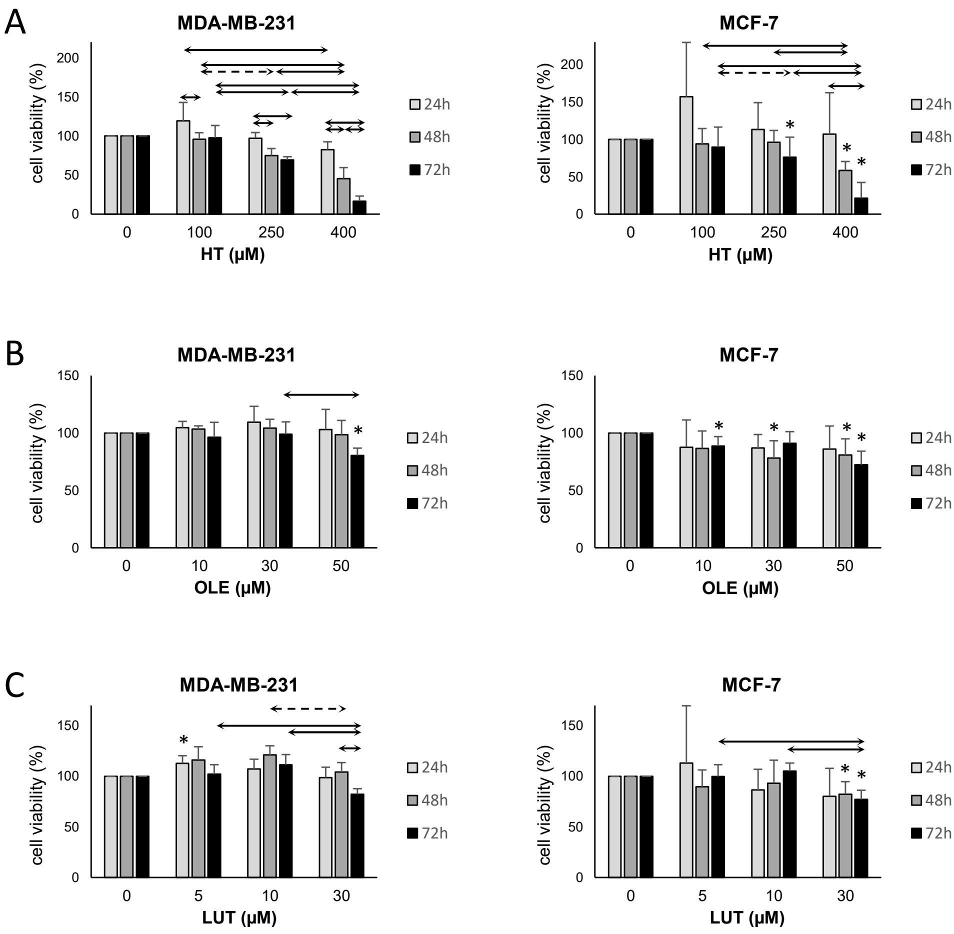

Figure S3. Effect of treatment with the EVOO minor compounds HT, OLE and LUT on cell viability in MDA-MB-231 and MCF-7 cell lines. Cell viability analysis using the MTT assay of MDA-MB-231 and MCF-7 cell lines treated with different doses of EVOO minor compounds for 24h, 48h or 72h. (**A**) Hydroxytyrosol (HT). (**B**) Oleuropein (OLE). (**C**) Luteolin (LUT). Mean + standard deviation of three independent experiments. *: $p < 0.05$ compared to control 0.1% DMSO-treated cells. Solid lines connecting bars indicate statistically significant differences ($p < 0.05$) between doses or time of exposure, dashed lines indicate differences close to significance ($p < 0.1$).

A

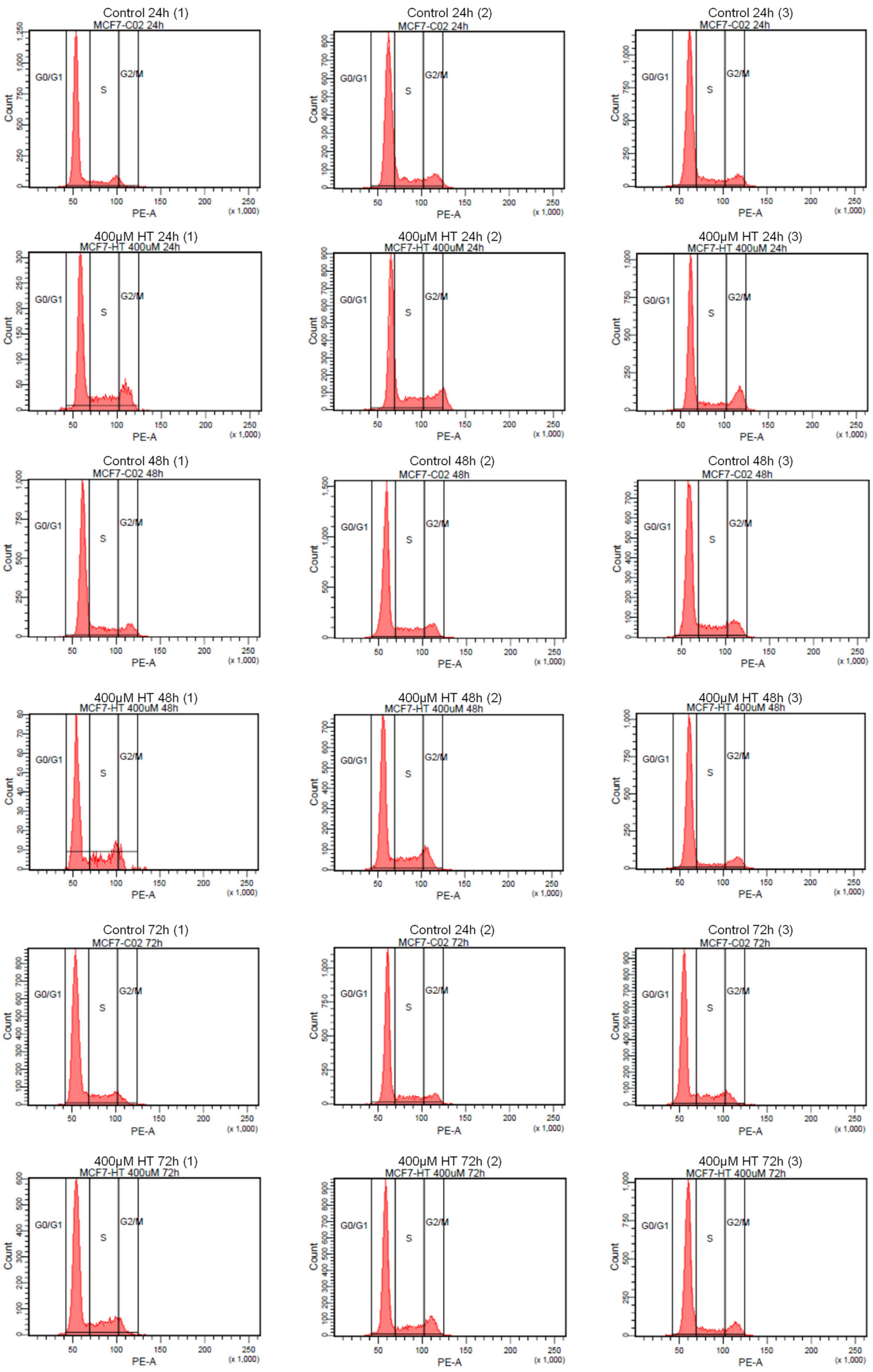
Control 24h (1)
MCF7-C02 24h
Control 24h (2)
MCF7-C02 24h
Control 24h (3)
MCF7-C02 24h
400μM HT 24h (1)
MCF7-HT 400uM 24h
400μM HT 24h (2)
MCF7-HT 400uM 24h
400μM HT 24h (3)
MCF7-HT 400uM 24h
Control 48h (1)
MCF7-C02 48h
Control 48h (2)
MCF7-C02 48h
Control 48h (3)
MCF7-C02 48h
400μM HT 48h (1)
MCF7-HT 400uM 48h
400μM HT 48h (2)
MCF7-HT 400uM 48h
400μM HT 48h (3)
MCF7-HT 400uM 48h
Control 72h (1)
MCF7-C02 72h
Control 24h (2)
MCF7-C02 72h
Control 72h (3)
MCF7-C02 72h
400μM HT 72h (1)
MCF7-HT 400uM 72h
400μM HT 72h (2)
MCF7-HT 400uM 72h
400μM HT 72h (3)
MCF7-HT 400uM 72h
G0/G1
S
G2/M
Count
PE-A
(x 1,000)

B

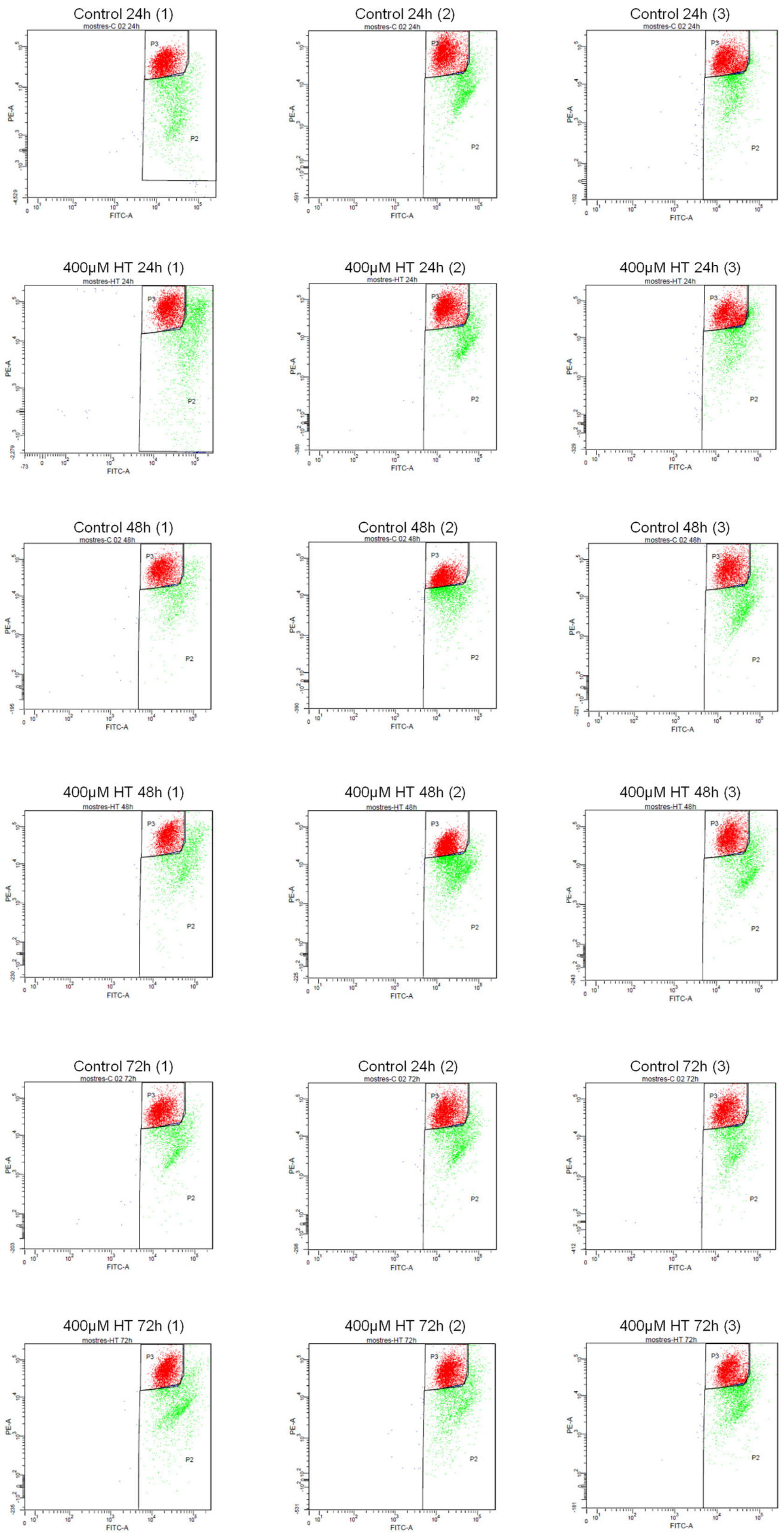

Figure S4. Original plots of the in vitro analysis of cell cycle and apoptosis by flow cytometry. (**A**) DNA content of cells stained with propidium iodide (G0/G1 and G2/M phase peaks are separated by the S-phase). (**B**) Dot plots of gating of JC1 aggregates (live cells, red fluorescence) and JC1 monomer (low mitochondrial membrane potential, apoptotic cells, green fluorescence) populations.
